# Low-level laser facilitates alternatively activated macrophage/microglia polarization and promotes functional recovery after crush spinal cord injury in rats

**DOI:** 10.1038/s41598-017-00553-6

**Published:** 2017-04-04

**Authors:** Ji Wei Song, Kun Li, Zhuo Wen Liang, Chen Dai, Xue Feng Shen, Yu Ze Gong, Shuang Wang, Xue Yu Hu, Zhe Wang

**Affiliations:** 1 Department of Orthopedics, Xijing Hospital, Fourth Military Medical University, Xi’an, Shaanxi China; 2 Department of Occupational and Environmental Health and the Ministry of Education Key Lab of Hazard Assessment and Control in Special Operational Environment, Fourth Military Medical University, Xi’an, Shaanxi China; 3 Department of Physics, Institute of Photonics and Photon-Technology, Northwest University, Xi’an, Shaanxi China

## Abstract

Macrophages and resident microglia play an import role in the secondary neuroinflammation response following spinal cord injury. Reprogramming of macrophage/microglia polarization is an import strategy for spinal cord injury restoration. Low-level laser therapy (LLLT) is a noninvasive treatment that has been widely used in neurotrauma and neurodegenerative diseases. However, the influence of low-level laser on polarization of macrophage/microglia following spinal cord injury remains unknown. The present study applied low-level laser therapy on a crush spinal cord injury rat model. Using immunofluorescence, flow cytometry, RT-qPCR, and western blot assays, we found that low-level laser therapy altered the polarization state to a M2 tendency. A greater number of neurons survived in the pare injury site, which was accompanied by higher BBB scores in the LLLT group. Furthermore, low-level laser therapy elevated expression of interleukin 4 (IL-4) and interleukin 13 (IL-13). Results from this study show that low-level laser therapy has the potential for reducing inflammation, regulating macrophage/microglia polarization, and promoting neuronal survival. These beneficial effects demonstrate that low-level laser therapy may be an effective candidate for clinical treatment of spinal cord injury.

## Introduction

The secondary neuroinflammation response is a key event after spinal cord injury (SCI)^1^. Previous studies have shown that modulation of this inflammation response can reduce the subsequent toxic effects. These methods include the depletion of macrophages with clodronate^2^ and delivery of anti-inflammatory chemicals to the spinal cord lesion site^3^. The overwhelming infiltration and activation of inflammatory cells induce a secondary cascade, and these cells release extensive cytokines, chemokines, and elicit events such as cell adhesion, migration, proliferation, cell death, and apoptosis. Haematogenous macrophage and resident microglia play important roles in sustaining neuroinflammation and impeding functional recovery^4–6^. However, a series of studies have shown that macrophages and microglia also play a favourable role^7, 8^. These contradictory functions might be due to their polarization states, which function as neurotoxic M1 phenotypes or reparatory M2 phenotypes^9–11^. The M1 subsets serve as the major population and predominate spinal cord lesions during the pathological process. Augmenting M2 or downregulating M1 has become an important strategy for SCI recovery^12–16^. Because macrophage/microglia polarization can be regulated by extensive inflammatory cytokines, studies have attempted to directly target the cytokines or indirectly achieve M2 polarization. A mouse SCI model was administered IL-6 receptor antibody, showing that blocking IL-6 signalling resulted in a greater M2 polarization ratio, increased expression of growth-associated protein (GAP)-43 and neurofilament heavy 200 kDa (NF-H), and increased locomotor scores^13^. A IL-10 lentivirus delivery model revealed that adoption of IL-10 drives the macrophage polarization in the M2 direction, which is maintained even if macrophages are exposed to pro-inflammatory stimuli^17^.

Low-level laser therapy (LLLT), also known as phototherapy or photobiomodulation (PBM), uses low doses of light from a laser. Low-level laser has been applied in several experimental disease models^18–20^ because of its ability to reduce inflammation. LLLT has been proposed to target mitochondrion and alter cellular responses through multilevel mechanisms, such as regulating the formation of reactive oxygen spices (ROS) and subsequently modulate transcription of several genes, including nuclear factor kappa B (NF-κB)^21^. Several studies have applied LLLT in transection and contusion SCI animal models^22–24^, which combined with our preliminary studies^25, 26^ proved that LLLT had beneficial effects on SCI rehabilitation, in particular inflammation alleviation. LLLT has also been applied to a transection SCI rat model to evaluate the biological characteristics of several inflammatory cells and cytokines^22^. The study applied a laser beam (810 nm wave length, 150 mW output power, 0.3 cm^2^ spot) focused on the surface of the skin and measured the penetration of the light to the spinal cord. It was confirmed that there was about 6% of total power transmitted and reached the ventral site of the spinal cord. The treatment was carried out for 14 consecutive days. The study applied retrograde and anterograde labeling methods to evaluate the regrowth of the axons. Results showed that LLLT has a significant effect on macrophage/microglia infiltration or activation during the early and sub-acute phase of SCI. The pro-inflammatory markers IL-6 and iNOS were also significantly suppressed by 161-fold and 5-fold, in the LLLT group, respectively. Additionally, laser therapy also improved the axonal regrowth and accelerated the locomotor recovery of the rats. Our former study^26^ also confirmed that LLLT elevates interleukin 10 (IL-10), but suppresses interleukin 6 (IL-6) and tumour necrosis factor-α (TNF-α) expression in the spinal cord lesion site in a contusion SCI model. Blockade of IL-6 signalling^13^ has also been shown to promote alternative activation of macrophages, and knockout of tumour necrosis factor (TNF)^12^ reduces persistence of M1 polarization. Therefore, we hypothesized that LLLT could also have a potential effect on macrophage polarization post-SCI. As far as we know, no other study has investigated the effect of LLLT on polarization of macrophages/microglia.

The present study used LLLT on a crush SCI model, showing that LLLT promoted alternative activation of macrophage/microglia. Results also showed that LLLT elevated expression of IL-4 and IL-13 in the spinal cord lesion site, suggesting that LLLT enhanced neuronal survival and promoted locomotor functional recovery.

## Result

### Macrophage/microglia polarization

**Figure 1:**
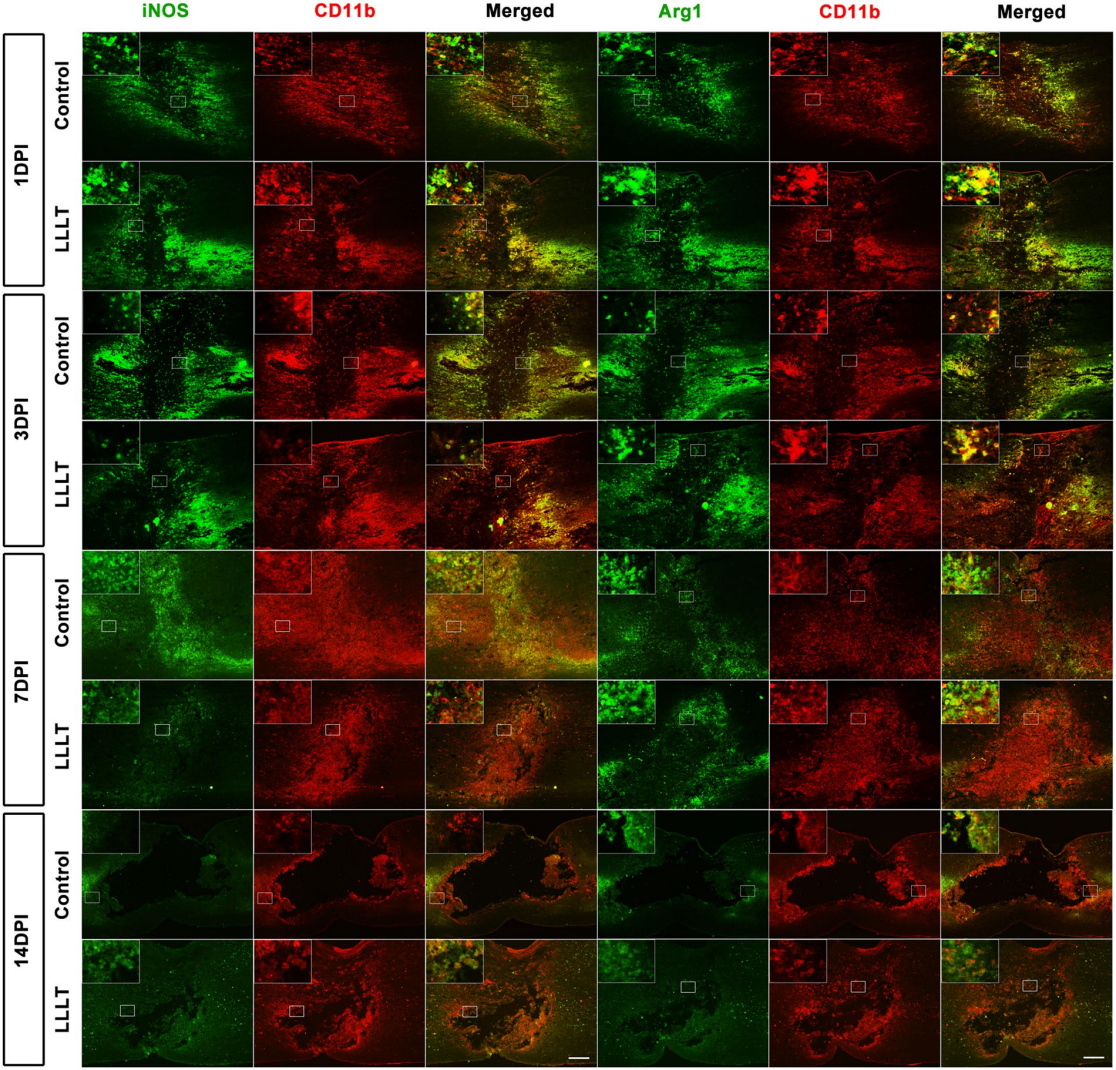
Time course of macrophage/microglia polarization. iNOS^+^ CD11b^+^ cells refer to a M1 phenotype, Arg1^+^CD11b^+^ cells refer to a M2 phenotype. Positive cells in the merged panels exhibit a yellow signal. The corner pictures are enlarged images of the small frame in each panel. Bar = 200 μm. iNOS, inducible nitric oxide synthase; Arg1, arginase 1. 1 dpi, 3 dpi, 7 dpi, and 14 dpi refer to 1, 3, 7, and 14 days post-injury, respectively.

**Figure 2:**
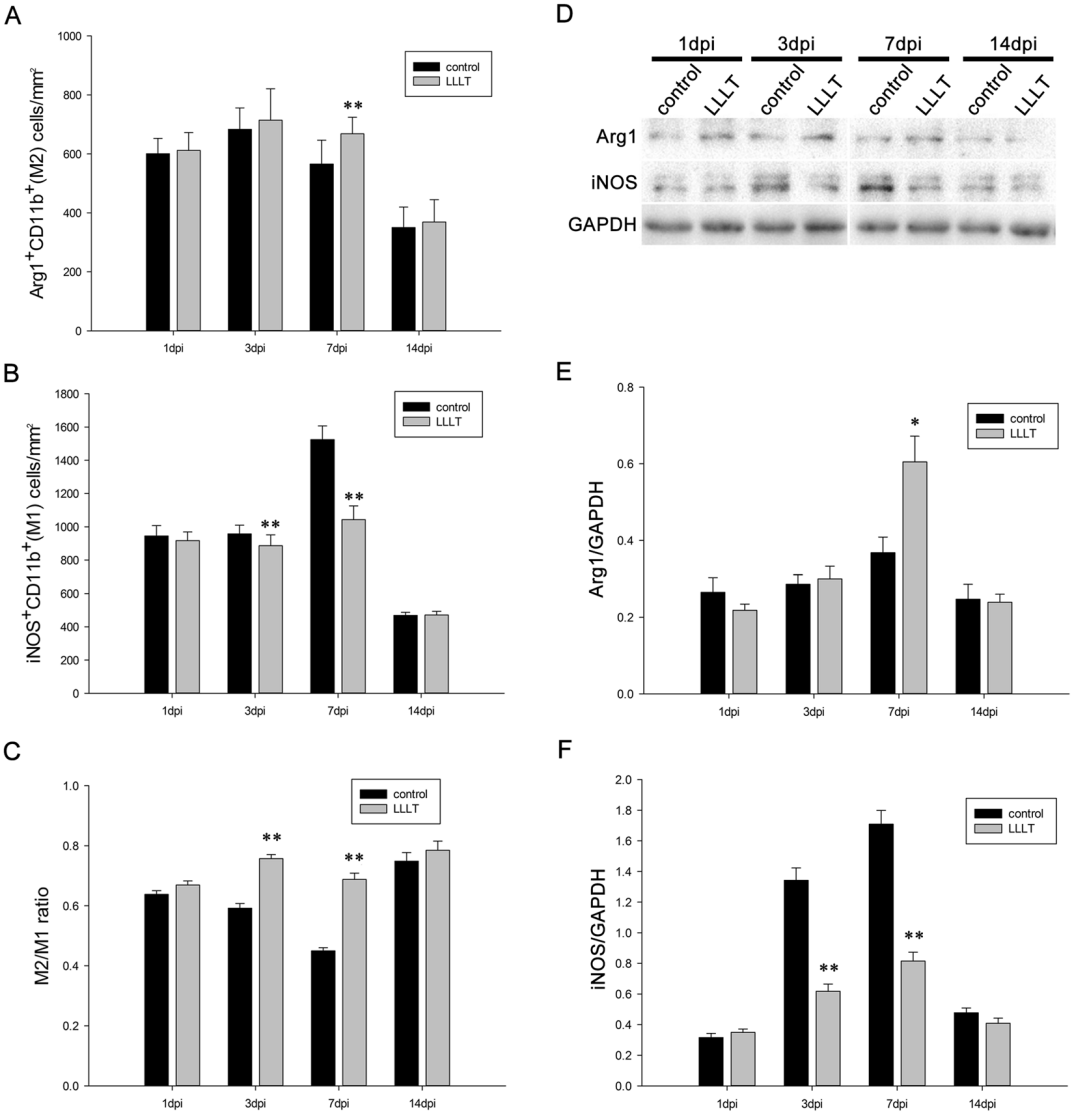
LLLT partly repels the M1 predominated polarization state and induces a relative elevated M2 expression. (**A**) M2 cell (Arg1^+^CD11b^+^) numbers per mm^2^ section compared between the two groups at different time points. (**B**) M1 cell (iNOS^+^ CD11b^+^) numbers per mm^2^ section compared between the two groups at different time points. (**C**) M2/M1 ratio at each time point. (**D**) Each band of the target protein (Arg1 and iNOS) and internal control (GAPDH) according to western blot analysis. (**E**) Time course of relative band density of Arg1. (**F**) Time course of relative band density of iNOS. 1 dpi, 3 dpi, 7 dpi, and 14 dpi refer to 1, 3, 7, and 14 days post-injury, respectively. (For IF: 5 measurements per section and 2 sections per rat with 3 rats per group; for WB: n = 3 rats per group *P < 0.05; **P < 0.01, compared with control).

**Figure 3:**
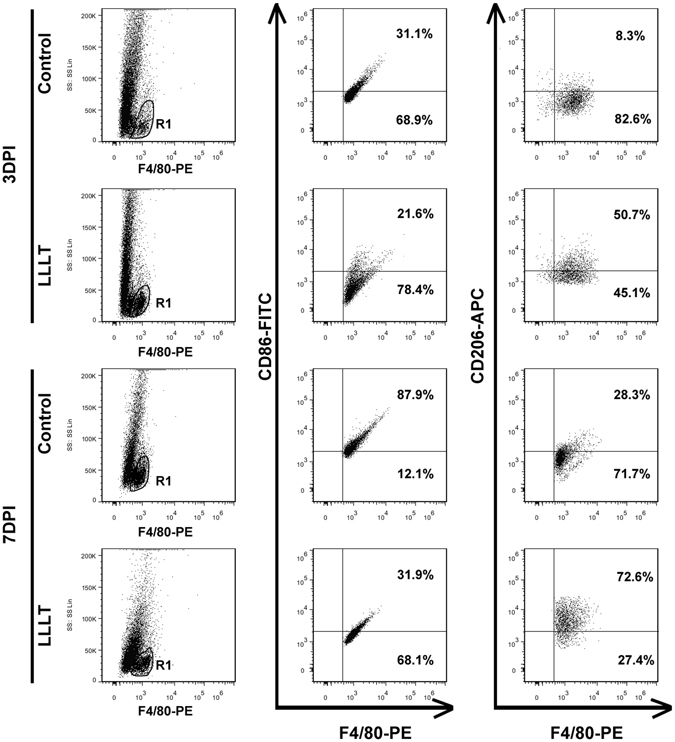
Dot plot of macrophage/microglia subpopulations. Representative dot plot of M1 and M2 subpopulations. R1 regions were gated as F4/80-positive populations. M1 cells were labelled with F4/80 and CD86, M2 cells were labelled with F4/80 and CD206. 3 dpi and 7 dpi refer to 3 and 7 days post-injury, respectively. SS, side scatter.

**Figure 4:**
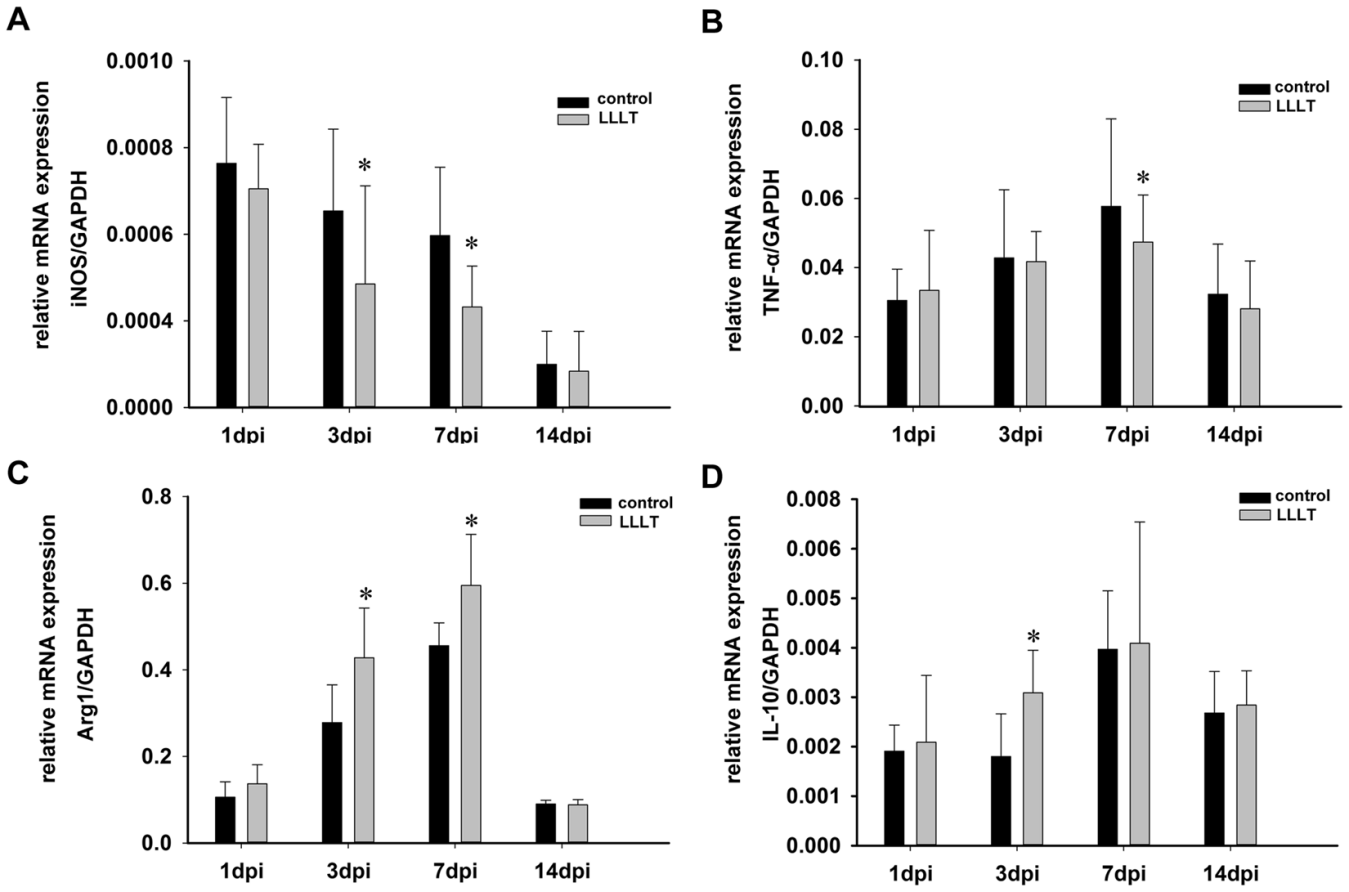
LLLT reduces mRNA expression of the M1 marker, but increases expression of M2. (**A**) Quantitative analysis of mRNA expression of M1 markers, iNOS. (**B**) Quantitative analysis of mRNA expression of M1 markers, TNF-α. (**C**) Quantitative analysis of mRNA expression of M2 markers, Arg1. (**D**) Quantitative analysis of mRNA expression of M2 markers, IL-10. iNOS, inducible nitric oxide synthase. Arg1, arginase 1. TNF-α, tumour necrosis factor-alpha. IL-10, interleukin 10. (n = 3 rats per group per time point *P < 0.05; compared with control).

Using sagittal sections, Immunofluorescence revealed that CD11b^+^ cells were mainly distributed in the lesion core and adjacent areas in the control and LLLT groups (Fig. 1). Of note, macrophage and microglia have been shown to be activated during the early stage of SCI^9, 27^, with activation continuing for substantial periods. A large number of CD11b^+^ cells emerged in the epicentre at 24 hours post-injury (Figs 1 and S2). The total area occupied by activated macrophage/microglia gradually increased from 1 day post injury (dpi) to 7 dpi and subsequently decreased at 14 dpi (Fig. 1). To investigate the effect of LLLT on macrophage/microglia polarization, double immunofluorescence study was performed. iNOS and Arg1 were used to label M1 and M2 phenotypes (Figs 1 and S1). Morphometric analysis revealed no significant difference in the number of iNOS^+^CD11b^+^ and Arg1^+^CD11b^+^ cells between control and LLLT groups at 1 and 14 dpi (Fig. 2A and B). However, the number of iNOS^+^CD11b^+^ cells significantly decreased in the LLLT group at 3 and 7 dpi (Fig. 2B). Conversely, the number of Arg1^+^CD11b^+^ cells significantly increased in the LLLT group at 7 dpi (Fig. 2A). The average number (per millimetre square cord section) of iNOS^+^CD11b^+^ cells in the control and LLLT groups was 957.3 and 886.0 at 3 dpi, and 1524.8 and 1043.3 at 7 dpi, respectively. The numbers of Arg1^+^CD11b^+^ cells were 565.8 in the control group and 668.3 in the LLLT group at 7 dpi. M2/M1 ratio assessment revealed a significantly higher ratio in the LLLT group at 3 and 7 dpi, respectively, compared with the control group (Fig. 2C). Western blot analysis coincided with these results; quantitative analysis revealed iNOS protein levels of 1.34 ± 0.11 and 1.79 ± 0.16 (vs. GAPDH) at 3 and 7 dpi, respectively in the control group. Compared with the control group, iNOS levels decreased by 1.66- and 2.09-fold in the LLLT group at 3 and 7 dpi, respectively (Fig. 2F). Concurrently, Arg1 protein expression was 0.65 ± 0.06 in the LLLT group, which was elevated by 1.64-fold, at 7 dpi (Fig. 2E). To further analyse M1 and M2 polarization, flow cytometry assay was performed. As Fig. 3 shows, the M1 fraction labelled by CD86 was reduced by LLLT at 3 and 7 dpi, respectively. Conversely, the M2 fraction significantly increased in the LLLT group. We performed RT-qPCR to identify mRNA expression of M1 and M2 markers in the two groups. Quantitative analysis revealed that LLLT significantly decreased mRNA expression of the M1 markers iNOS (at 3 and 7 dpi) and TNF-α (at 7 dpi). Conversely, LLLT significantly increased expression of the M2 marker IL-10 (at 3 dpi) and Arg1 (at 3 and 7 dpi) (Fig. 4). These results suggested that although the lesion site was mainly occupied by iNOS^+^CD11b^+^ cells, LLLT partly reversed the M1 predominant state and drove polarization to a relatively M2 elevated state at 7 dpi.

### Inflammation response

**Figure 5:**
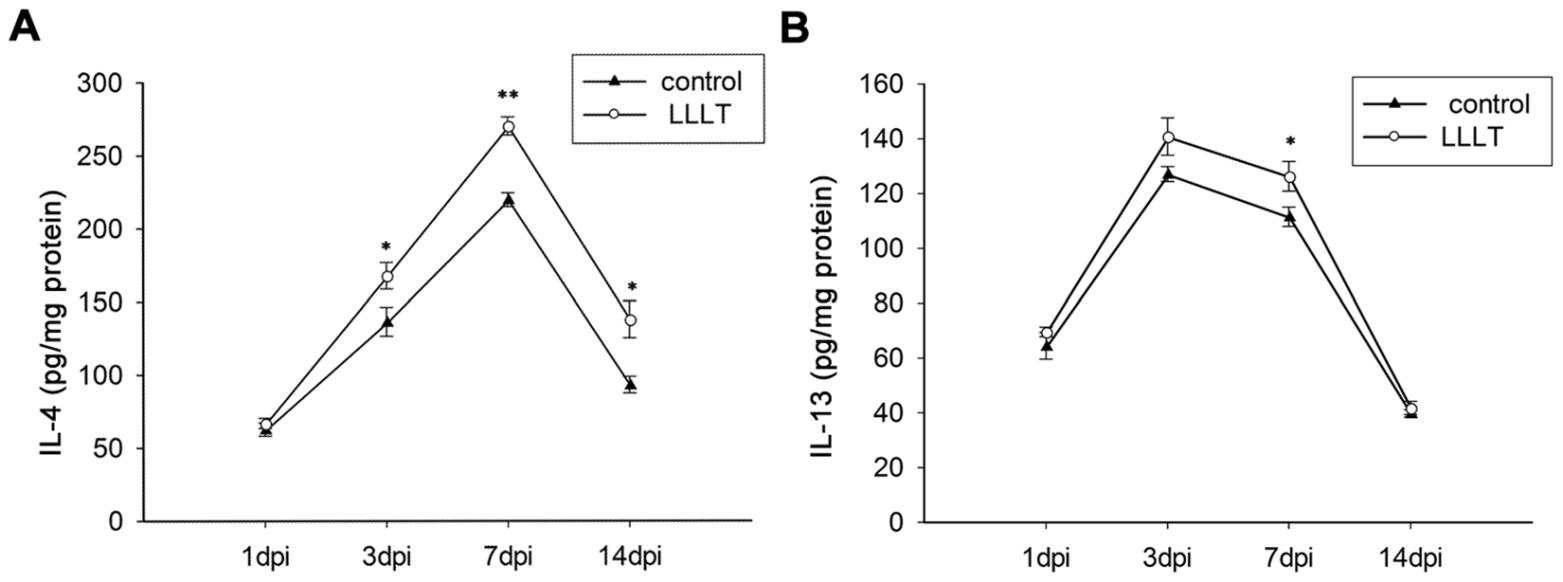
LLLT increases IL-4 and IL-13 expression. (**A**) Time course of IL-4 relative expression in control and LLLT groups. (**B**) Time course of IL-13 relative expression in control and LLLT groups. IL-4, interleukin 4. IL-13, interleukin 13. (n = 3 rats per group *P < 0.05; **P < 0.01, compared with control).

ELISA was performed to analyse IL-4 and IL-13 expression in the LLLT and control groups. As shown in Fig. 5, IL-4 expression gradually increased over time, with a maximum at 7 dpi. Peak IL-13 expression was observed at 3 dpi. LLLT resulted in significantly increased IL-4 expression at 3, 7, and 14 dpi compared with the control group. IL-4 total protein concentrations in the LLLT group were 167.8 pg/mg, 270.3 pg/mg, and 137.9 pg/mg at 3 dpi, 7 dpi, and 14 dpi, respectively. In contrast, concentrations in the control group were 136.4 pg/mg, 219.7 pg/mg, and 93.2 pg/mg, respectively. IL-13 protein elevation was only observed at 7 dpi in the LLLT group. These results suggested that LLLT regulated IL-4 and IL-13 expression, thereby creating a preferential environment for M2 polarization.

### Neuroprotection

**Figure 6:**
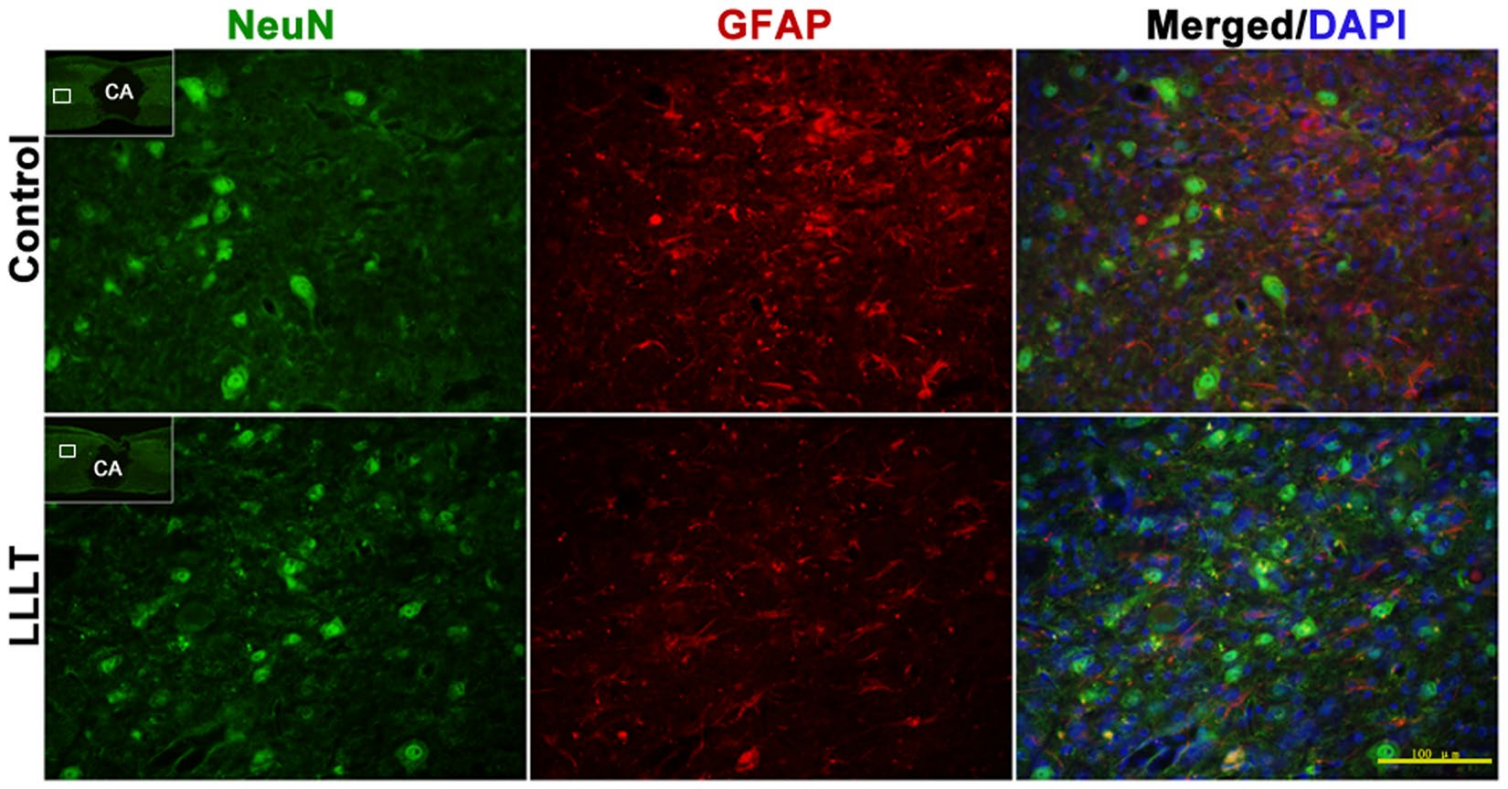
Neurons and astrocytes coexist in the para injury site. Representative immunostaining images of neurons (NeuN, green signal) and astrocytes (GFAP, red signal). Images were obtained from para injury site. The images are enlarged pictures of the small quadrilateral region in the sections. NeuN, neuronal nuclei. GFAP, glial fibrillary acidic protein. CA, cavity. Bar = 100 μm.

**Figure 7:**
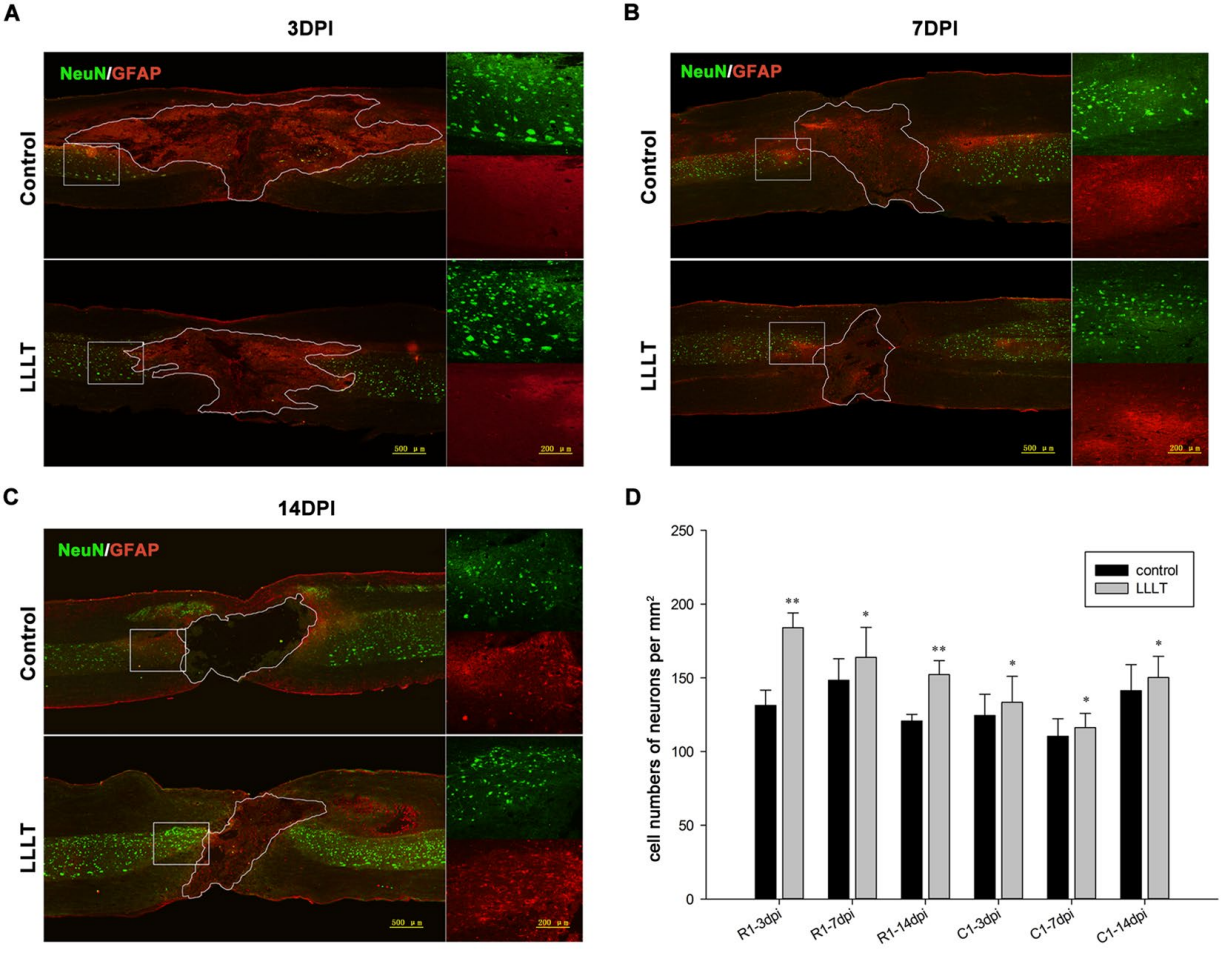
LLLT increases neuronal survival. (**A–C**) Representative double-immunostaining images of neurons and astrocytes at 3, 7, and 14 dpi. The epicentre of the section is outlined in accordance with GFAP expression. Right images in each panel are enlarged images of the boxed area rostral to the epicentre. (**D**) Quantitative analysis of neurons in regions 500 μm rostral (R1) and 500 μm caudal (C1) to the epicentre. Neuronal cell number was averaged and presented as cell number per mm^2^ cord section. NeuN, neuronal nuclei. GFAP, glial fibrillary acidic protein. In the left part of panel A, B, and C, bar = 500 μm. In the enlarged images, bar = 200 μm. (R1 or C1: 5 measurements per section and 2 sections per rat with 3 rats per group; *P < 0.05; **p < 0.01, compared with control).

We further investigated the effect of LLLT on neuronal survival. NeuN staining revealed a loss of neuronal cells in the epicentre following SCI. Double staining revealed that reactive astrocytes coexisted with surviving neurons at 14 dpi (Fig. 6). However, the lesion adjacent area was predominately occupied by GFAP^+^-labelled reactive astrocytes at 7 and 14 dpi (Fig. 7B and C). Neuronal quantification was performed to assess general neuronal cell loss in the para injury site. The total number of surviving neurons in the R1 (500 μm rostral to the epicentre) and C1 (500 μm rostral to the epicentre) region was significantly greater in the LLLT group at 3, 7, and 14 dpi (Fig. 7D). These results indicated that LLLT has a beneficial neuroprotective effect in the SCI environment.

### Lesion area and cavitation

**Figure 8:**
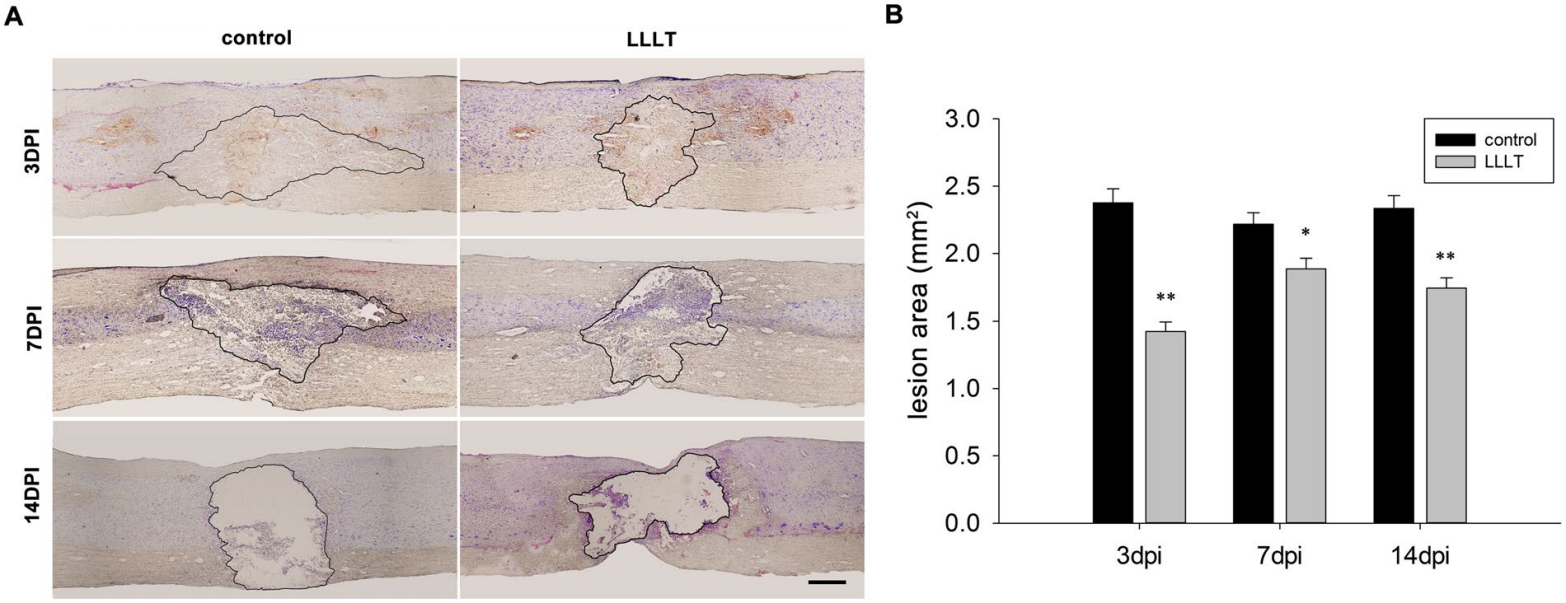
LLLT shrinks the SCI lesion area. (**A**) Representative images of lesion profile and cavitation in control and LLLT groups at 3, 7, and 14 dpi, respectively. The lesion area and cavity are outlined according to Nissl staining with cresyl violet. Lesion assessment was location-matched between the two groups. (**B**) Assessment of lesion and cavity area at 3, 7, and 14 dpi. Bar = 500 μm. (n = 2 sections per rat with 3 rats per group; *P < 0.05; **P < 0.01, compared with control).

We next explored the effect of LLLT on lesion profile and cavitation. The lesion area was measured in sagittal sections stained with cresyl violet at 3, 7, and 14 dpi. The representative lesion profile of each group is presented in Fig. 8A. Nissl staining showed a loss of neural cells in the epicentre early after injury. In the lesion epicentre, neural cells and tissue loss amplification occurred in a time-dependent manner. Ultimately, the cavity emerged and grey matter collapsed, with a small region of white matter reserved in the epicentre. Quantitative analysis revealed a significantly reduced average lesion area in the LLLT group at 3 and 7 dpi, and the cavity area was also reduced by LLLT at 14 dpi (Fig. 8B).

### Behaviour evaluation

**Figure 9:**
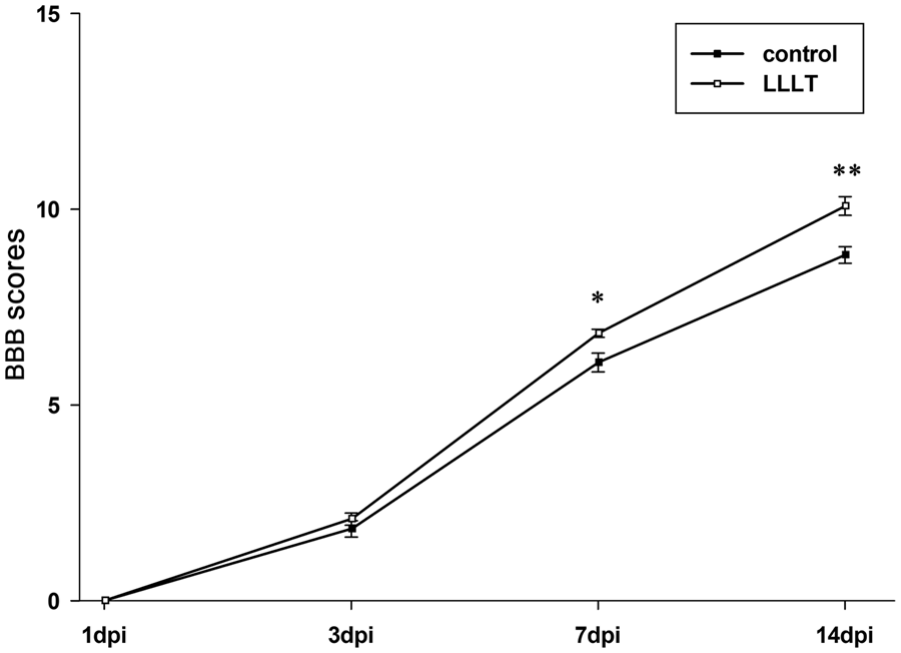
LLLT promotes locomotor recovery in SCI rats. BBB scores, Basso–Beattie–Bresnahan scores. (Data are presented as mean ± SEM for n = 6 rats per group; *P < 0.05; **P < 0.01, compared with control).

The Basso–Beattie–Bresnahan (BBB) locomotor rating scale is a convenient and widely used method for functional recovery evaluation in rat SCI models. The scale was designed to assess locomotion by monitoring behaviour of the hind limbs, such as joint movement, paw placement, coordination, stepping, and tail position. In the present study, scores in both groups declined to zero post-surgery. At 3 dpi, the scores increased to around 3 points in both groups, with no significant difference between groups. However, BBB scores in the LLLT group were significantly greater at 7 and 14 dpi compared with the control group (Fig. 9). These results suggested that rats achieved better locomotor function recovery following laser therapy.

## Discussion

Results from the present study demonstrated that LLLT had a moderate influence on macrophage/microglia polarization. LLLT partially repelled the M1 “bias” and significantly decreased iNOS expression. Moreover, this treatment drove the polarization state to a relatively increased M2 state during the sub-acute phase of SCI. LLLT alleviated the inflammation response and significantly increased IL-4 and IL-13 expression. Finally, treatment reduced neuronal cell loss and the size of the lesion area in the spinal cord.

The secondary cellular neuroinflammatory response, in particular spatiotemporal macrophage/microglia activation post-SCI, has been extensively described^28–30^. However, these studies did not look at the effect of LLLT. Previous studies showed that due to a disrupted blood-spinal cord barrier, clusters of peripheral macrophages infiltrate into the lesion site post-injury^31, 32^. Haematogenous macrophages migrate to the lesion site because of several pairs of chemokines and their ligands^33^. Spinal cord resident microglia become activated and alter their morphological character within minutes after injury. Spatially, haematogenous macrophages are mainly distributed in the lesion core, while resident microglia occupy the para injury site^34^. Temporally, the number of these activated cells reached a peak in the present study between 7 and 14 dpi, but then subsequently declined, although some remained in the spinal cord for months. LLLT was applied to the SCI lesion site for 14 consecutive days post-injury. Morphological analysis showed that although LLLT did not alter the general spatiotemporal tendency of macrophage/microglia activation, the total number of CD11b^+^ cells significantly decreased following LLLT at 3 and 7 dpi (Fig. S2). This finding was consistent with a previous study^22^. Because the main contribution of the inflammatory process is likely due to macrophage/microglia, it is important to note that LLLT reduced the comprehensive inflammation response of SCI. Future studies should focus on the mechanisms of adhesion, infiltration, and activation of macrophages in response to LLLT.

Infiltrated macrophages and resident microglia can be divided into two main subsets due to functional and cell marker distinctions. The predominant state of M1 leads to an extensive inflammation response and contributes to damage expansion. Unfortunately, the M1 subset is predominant and sustained, while the M2 subset is minor and transient in SCI pathology^9, 12^. The present immunofluorescence study revealed significantly increased numbers of CD11b^+^iNOS^+^-labelled M1 subsets at 3 and 7 dpi, which continuously dominated the lesion site in the control group. The number of M2 subsets reached a peak between 3 and 7 dpi, which then subsequently declined. In the LLLT group, although M1 subsets were still the main subpopulations, the peak number was significantly smaller at 3 and 7 dpi. These results showed that LLLT reduced the total number of M1 subpopulations during the early phase of SCI. This suppression could be a secondary effect of a reduced total number of macrophage/microglia elicited by LLLT. However, LLLT also up-regulated the M2 subpopulation at 7 dpi, which resulted in a significantly increased ratio of M2/M1. This demonstrated that LLLT influenced phenotype transduction at 7 dpi. The neurotoxic M1 subset secretes numerous Th1 cytokines, such as TNF-α, IL-1, and IL-6^35^. Conversely, the neuroprotective M2 delivers diverse Th2 cytokines, such as IL-10, IL-4, and TGF-β, as well as some neurotropic factors^36^. Numerous studies designed to alter the intractable polarization status to a M2 tendency have demonstrated better SCI recovery^13, 15, 16, 37^. Our findings provided evidence that LLLT regulated the biological activity of macrophages and modulated the polarization state to a M2 tendency during the sub-acute phase of SCI. This may be one of the beneficial effects of LLLT that contribute to locomotor recovery in rats.

LLLT has been shown to create changes in a dose- and time-dependent manner^38, 39^. Therefore, we applied LLLT to the spinal cord lesion site within 30 minutes post-injury and continued treatment for 14 consecutive days. This treatment was expected to modulate not only the early influx of peripheral macrophages, but also the continuous predomination of M1 macrophage/microglia. However, results showed that LLLT did not improve the M2 fraction throughout treatment. A former study revealed that LLLT had no significant influence on T or B lymphocytes, but did alter the number of macrophages and neutrophils^22^. The pathology process of SCI resulted in an obvious reduction of activated macrophages/microglia, although this inflammatory response significantly decreased by 14 dpi. Results showed that the overall inflammatory modulation effect elicited by LLLT did not alter the imbalance of macrophage/microglia polarization at 14 dpi. LLLT may also not have a significant effect at 1 dpi.

The dynamic phenotype expression of macrophage/microglia was affected by the neuroinflammatory environment following SCI. The sustained high levels of pro-inflammatory cytokines contributes to a persistent M1 polarization^40^. It has been suggested that macrophage/microglia could be polarized in response to different stimuli, such as cytokine clusters^17, 41–43^. IL-4 and IL-13 are anti-inflammatory cytokines that are commonly used for alternative polarization of the Raw264.7 macrophage cell line. IL-4 and IL-13, as well as IL-4R and IL-13R, signalling are essential for this polarization network^44^; they activate downstream transcription of the signal transducer and activator of transcription 6 (STAT6) and promote M2 polarization. They are also important modulators of the SCI inflammation response. Neutralization of IL-4 has also been shown to elicit an extension of macrophage activation^42^. In the present study, LLLT elevated IL-4 expression at 3, 7, and 14 dpi and elevated IL-13 expression at 7 dpi. These expression changes were similar to the increased tendency towards M2 cells. Results suggested that IL-4 and IL-13 could participate in the up-regulation of M2 cells modulated by LLLT. Interestingly, although IL-4 expressions were elevated at 3 and 7 dpi, the ratio of M2 cells significantly increased only at 7 dpi, which was consistent with the tendency towards IL-13 expression. These results suggested that IL-13 may have an important synergistic effect with IL-4 in the up-regulation of M2 cells. Our former study^26^ and the study by Byrnes^22^ showed that LLLT regulates several cytokines after spinal cord injury, including suppression of the pro-inflammatory cytokines TNF-α, interleukin 1β (IL-1β), and IL-6, as well as up-regulation of the anti-inflammatory cytokine IL-10. It is possible that these cytokine level changes modulated the microenvironment and drove the polarization state towards a M2 direction. LLLT may elicit M2 signalling and reprogram the polarization towards a reparatory direction. However, the detailed mechanisms modulated by LLLT require further investigation.

Neuronal cell loss is a disastrous secondary damage following SCI, which leads to neurological deficits^45, 46^. The amelioration of this phenomenon allows for some rehabilitation in SCI animal models^47–49^. In the present study, interestingly, the number of surviving neurons significantly increased in the LLLT group in the rostral and caudal side, suggesting that LLLT has a general protective effect on neurons after injury. We also discovered that the spatial and temporal distribution of surviving neurons was distinct between the two groups. The number of surviving neurons in rostral side exceeded those in the caudal side at 3 and 7 dpi in the LLLT group. However, at 14 dpi, the number of surviving neurons was similar in both sides. This phenomenon was observed only at 7 dpi in the control group. The differences in spatial distribution of surviving neurons between the two groups suggest that LLLT has a stronger protective impact on the rostral side. The neuronal protective effect began at 3 dpi, which correlated with a reduction in M1 cells. These findings suggested that the protective effect might may in part due to the reduced number of M1 cells. M2 macrophages/microglia function as an anti-inflammatory subset, expressing high levels of cytokines such as IL-10 and TGF-β, as well as neurotrophic factors such as nerve growth factor (NGF)^50^ and insulin-like growth factor-1 (IGF-1)^51^. These beneficial cytokine and neurotrophic factors participate in neuronal protection and survival. The protective effect in our study was detected out to 14 dpi, which also coincided with the period of M2 cell elevation. These results suggested that the change in M2 cells also contributed to the neuroprotective effect. Although the total lesion area at 14 dpi was larger than or equal to 3 dpi in general, LLLT reduced the cavity area compared with the control group (Fig. 8). Histological images showed that the haemorrhage area, which was also in the para injury site, spread far away from the epicentre and differed among individuals (data not shown) at 3 dpi. We assumed that the major portion of the injury area was still reserved at this time point. At a later stage, comprehensive tissue damage area can transform into necrosis and gradually form a cavity. This could help to explain the phenomenon. We also deduced that the combined effect of reduced M1 toxicity and enhanced neuronal survival contributed to a decreased lesion area and the acceleration of neurological recovery, as indicated by higher BBB scores.

Collectively, our study showed that LLLT promoted structural and functional recovery in a crush SCI model. LLLT drove polarization of macrophage/microglia towards a M2 direction, and more importantly, this modulation effect correlated with enhanced neuronal survival and a reduction in lesion area. These results provide preliminary evidence that LLLT could serve as an effective treatment candidate for SCI patients in the future.

## Methods

### Animals and Ethics statement

96 Sprague Dawley rats weighed 220~260 g, were obtained from the animal center of Fourth Military Medical University, Xi’an, China. Animals were kept in clean and warm cages under a 12-h light/dark circle with food and water supply ad libitum. Animals were randomly assigned to surgery only group (control) or surgery followed by laser irradiation group (LLLT), with 48 rats per group. All experiments were conducted in strict compliance with the guidelines established by the Animal Care and Use Committee of the Fourth Military Medical University. The protocol was approved by the Animal Care Ethics Committee of the Fourth Military Medical University. Necessary cares were taken to minimize the animal sacrifice and suffering.

### Surgery and animal care

A bilateral spinal cord compression model was performed according to the former studies^52, 53^ with slight modification. Briefly, after animals were anesthetized (1% sodium pentobarbital, 50 mg/kg, intraperitoneally) a longitudinal incision above T7~T10 vertebra was made. Then a dorsal laminectomy was performed at T8 to expose the spinal cord. Lateral compression was performed with a pair of forceps (Fine Science Tools, Heidelberg, Germany) which were modified by adding a metal spacer between the blades to attain a 0.5 mm gap when fully closed. The blades of the forceps were placed vertically to the vertebral canal and kept maximally closed for 20 seconds then released. Hemostasis was made and the incision was closed in layers. Rats in the whole procedure were placed on a homothermal blanket to maintain body temperature at around 37 °C. Manual bladder squeeze was performed twice daily until the urination of animal returned.

### Laser irradiation

Laser irradiation was applied in percutaneous method according to the former study^22^. Rats were slightly anesthetized and put into a warm cage in dark, a continuous 810 nm diode laser beam (MW-GX-808, Lei Shi Optoelectronics Co., Ltd. Changchun, China, 810 nm wavelength, 150 mW output power, 0.3 cm^2^ light spot) was used percutaneously in vertical direction focus on the injury site of the spinal cord. Laser treatment was continued for 50 minutes per rat daily for a total of consecutive 1 to 14 days in LLLT group. The irradiation parameters have been identified safe and the energy could be effectively transmitted to the surface and the depth of the spinal cord^22^. Rats in SCI group were treated equally except laser application.

### BBB test

Basso–Beattie–Bresnahan (BBB) scale^54^ was used for locomotor function evaluation as has been described. Rats were allowed to be acclimated to the test field once daily for 3 days before surgery. Before tests, bladders of the rats were manually squeezed. Then the animals were put into a 100 cm × 100 cm transparent Plexiglas box with walls at 10 cm height and observed for a total 4 minutes per rat. Each animal was evaluated independently by two observers who were blind to animal grouping. The average score was used for statistical analysis.

### Tissue preparation

At the designed time points of the experiment (1, 3, 7, 14 days post injury; 1 dpi, 3 dpi, 7 dpi, 14 dpi), 12 animals each group were randomly selected to accomplish the following experimental procedures, of which 3 rats were sacrificed for immunofluorescence (IF) study, 3 rats for western blotting (WB) and enzyme linked immunosorbent assay (ELISA), 3 rats for Reverse transcription quantitative real-time PCR (RT-qPCR), the rest 3 rats for flow cytometry (FC) experiment. For IF, rats were perfusion with paraformaldehyde in phosphate buffer (4%, 4 °C, pH 7.4) and 2 cm long spinal cord segment centering at the injury site was carefully dissected and put into same fixation solution within 4~6 h. Then the cord was transferred into 25% glucose in phosphate buffer at 4 °C till the tissue sank. Afterward, 14 μm serial sagittal sections was cut on a cryostat (CM1900, Leica, Germany) and mounted on superfrost slides for 10 sets in a cyclic pattern. For protein preparation of WB and ELISA, 5 mm tissue segment with the injury site at middle was dissected. The weight of each tissue was measured and recorded. Then the segment was put into ice cold lysis buffer and homogenized by a tissue homogenizer on ice. The samples were centrifuged for 10 minutes (4 °C, 13000 rpm), then the supernatants were collected and transferred to a new Eppendorf tube and stored at −80 °C till use. For RT-qPCR, the mRNA expression was measured by real-time reverse transcription PCR. At 1, 3, 7, 14 days after crush injury, total RNA of spine cord was extracted from LLLT group and control group (5 mm spinal cord segment containing the injury epicenter) using total RNA extraction kit (Omega Biotech, USA). For flow cytometry, the crushed portion of each spinal cord (5 mm around the epicenter) was carefully dissected. Then the sample was dissociated with collagenase (175 U/mL; Sigma-Aldrich) for 1 h at 37 °C. Afterward the cells were washed in Dulbecco’s modified Eagle’s medium (Invitrogen Life Technologies, Carlsbad, CA) containing 10% fetal bovine serum, To remove tissue debris and obtain a single-cell suspension, the cells were filtered through a 40-μM nylon cell strainer under centrifugation (5 mins; 1000 rpm).

### Nissl’s staining and lesion area measurement

Lesion area measurement was performed in cresyl violet stained longitudinal sections^16^. The frozen sections were incubated with 1% cresyl violet (Beyotime, Shanghai, China) according to the instructions given by the manufacture. Measurement was location-matched between the two groups. The borderline of the lesion core were recognized and the lesion area was measured with Adobe Photoshop CS5 (Adobe, San Jose, USA).

### Immunofluorescence

Initially, sections were rinsed in phosphate buffer saline (PBS) for 5 minutes thrice. Then the sections were blocked in 1% donkey serum containing 0.3% triton X-100 for 30 minutes at room temperature. Afterward sections were incubated with the primary antibodies overnight at 4 °C and incubated with appropriate secondary antibodies for 2 hours at 37 °C. Finally, sections were counterstained with 4′, 6-diamidino-2-phenylindole (DAPI) to label nuclei and coversliped. Images were taken under a fluorescence microscopy (BX51, Olympus, Tokyo, Japan). The following primary antibodies were used: mouse anti-glial fibrillary acidic protein (GFAP, 1:1000, Abcam plc, Cambridge, UK), rabbit anti-neuronal nuclei (NeuN, 1:500, Abcam plc, Cambridge, UK), mouse anti-integrin αM (CD11b, 1:1000, AbD Serotec, Kidlington, UK), rabbit anti-inducible nitricoxide synthase (iNOS, 1:100, Santa cruz, Texas, USA), rabbit anti-Arginase 1 (Arg1, 1:100, Santa cruz, Texas, USA). The corresponding Alexa Flour® fluorescent secondary antibodies were purchased from Abacm plc, Cambridge, UK.

### Macrophage/microglia polarization assessment

Macrophage/microglia subsets counting were performed in iNOS^+^CD11b^+^ (recognized as M1) or Arg1^+^CD11b^+^ (recognized as M2) double labeled sections in the lesion adjacent area (500 μm caudal and rostral to the epicenter). Two sections adjacent to the central canal were selected. These two sections distributed approximately 150 μm laterally to the central canal, contained the main portion of the gray matter. Target cells were confirmed due to the CD11b exhibited a circular staining profile at the cell membrane and iNOS or Arg1 positive staining located in cytoplasm. Positive stained cells only with clear nucleus counterstained with DAPI were included in cell counting. For each selected section, cell counting was performed in 5 random 400× high power magnification fields with a total of 10 fields per rat. Number of cells was normalized to per millimeter square cord section.

### Flow Cytometry

Immunostaining was performed following standard protocols and 1% bovine serum albumin (BSA) was used to block nonspecific antibody binding. PE-CD11b (0.25 μg: 10^6^cells in 100 μl volume; BioLegend, San Diego, CA, USA) was used to mark macrophage/microglia; FITC-CD86 (1.0 μg: 10^6^cells in 100 μl volume, Biolegend, San Diego, CA, USA) and APC-CD206 (0.2 μg: 10^6^cells in 100 μl volume, Biolegend, San Diego, CA, USA) were used to mark M1 and M2 subpopulations. Cells were analyzed using a Beckman F500 flow cytometer, at least 10,000 cells were analyzed each sample. The results were analyzed using FlowJo software ((TreeStar, San Carlos, CA, USA).

### Reverse transcription quantitative real-time PCR

Total RNA was transcribed into cDNA using a reverse transcription system (TakaRa, Kyoto, Japan). Each step was performed according to the manufacturers’ instructions. Real-time PCR was performed on an CFX96 Touch™ Real-Time PCR Detection System (Bio-Rad Laboratories, CA, USA) using PrimeScript™ RT Master Mix (TaKaRa, Kyoto, Japan). Glyceraldehyde-3-phosphate dehydrogenase (GAPDH) housekeeping gene was used to normalize gene expression by parallel amplification. The relative expression level of target mRNAs was calculated. PCR primer sequences are listed in Table 1.

**Table 1:** Sequences of primers used for real time qPCR experiments.

Gene	Forward primer 5′-3′	Reverse Primer 5′-3′
TNF-α	CACGCTCTTCTGTCTACTGAACTTCG	TGCTCCTCCGCTTGGTGGTT
iNOS	ATCTTGGAGCGAGTTGTGGATTGTT	GGTAGTGATGTCCAGGAAGTAGGTGA
IL-10	GGGTTGCCAAGCCTTGTCAGAAA	CTTCACCTGCTCCACTGCCTTG
Arg-1	TGTGGTAGCAGAGACCCAGAAGAAT	CAGCGGAGTGTTGATGTCAGTGT

### Neuronal cell counting

Neuronal cell counting was performed in GFAP/NeuN double stained longitudinal sections at 3, 7, 14 dpi. Cell counting was location-matched between the two groups. Initially, the boundary of GFAP strong positive area was outlined, then 5 random 400× high power magnification fields in the region 500 μm rostral (defined as R1 region) to the boundary were selected. 5 fields within the region 500 μm caudal (defined as C1 region) were also analyzed. The total number of neurons in R1 and C1 were counted with NIH Image J software (NIH, Bethesda, USA) and averaged respectively. Number of neurons was normalized to per millimeter square cord section.

### Western blot

The total protein of each sample was measured with BCA Protein Assay Kit (Thermo Scientific, Rockford, USA). A total 30 μg protein from each sample was separated in running buffer and transferred to a polyvinylidenedifluoride membrane (Millipore, Billerica, USA). The membrane was blocked in 5% defatted milk for 1 h at room temperature and incubated overnight at 4 °C with the primary antibodies: Rabbit anti-iNOS(1:500, Santa cruz, Texas, USA.), Rabbit anti-Arg1(1:500, Santa cruz, Texas, USA), Mouse anti- glyceraldehyde phosphate dehydrogenase (GAPDH, 1:1000, abcam plc, Cambridge, UK). Thereafter, the membrane was rinsed in Tris-buffered saline with Tween-20 (TBST) for 10 minutes at three times, followed by HRP-conjugated secondary antibody incubating for 1 h at room temperature. The immunoreactive bands were visualized with ECL Western Blot Kit (Thermo Scientific, Rockford, USA). The bands were scanned and digitalized. The density of each band was measured with Image Lab 4.1 software (Bio-rad, CA, USA). The percentage of target protein’s band density to that of GAPDH was calculated and used for statistical analysis.

### Enzyme linked immunosorbent assay

The samples were diluted to a concentration suggested by the ELISA kit manufacture (Boster, Wuhan, China). The samples, interleukin 4(IL-4) and interleukin 13(IL-13) standard protein were prepared in duplicates, the immunoreaction procedure was performed according to the protocol provided by the ELISA kit manufacture. Absorbance of the wells was measured at a wavelength of 450 nm by a spectrophotometer (Shanghai Spectrophotometer Co. Ltd., China). Then the concentration of each sample (pg/ml) were calculated, and divided by the total protein concentration (mg/ml). The amount of IL-4 and IL-13 that present in per mg of total protein (pg/mg) in each sample was determined.

### Statistical analysis

All data was presents as Means ± SEM. Statistical analysis and graphics were established with SigmaPlot (Systat, Erkrath, Germany). Analysis between the two groups was performed with Student’s t tests. A level of p ≤ 0.05 was set as statistical significance.

## Electronic supplementary material

Supplementary Information
