# Supplementary material for: Low-level laser facilitates alternatively activated macrophage/microglia polarization and promotes functional recovery after crush spinal cord injury in rats
Source: Sci Rep. 2017 Apr 4;7:620. doi: 10.1038/s41598-017-00553-6 (PMC5428709; doi:10.1038/s41598-017-00553-6)
Supplement: Supplementary file 1 — Supplementary Information [file 41598_2017_553_MOESM1_ESM.pdf]

Supplementary Information

"Low-level laser facilitates alternatively activated macrophage/microglia polarization and promotes functional recovery after crush spinal cord injury in rats"

Ji Wei Song[1#], Kun Li [1#], Zhuo Wen Liang[1], Chen Dai[1], Xue Feng Shen[2], Yu Ze Gong[3], Shuang Wang[3], Xue Yu Hu[1*] & Zhe Wang[1*]

[1] *Department of Orthopedics, Xijing Hospital, Fourth Military Medical University, Xi'an, Shaanxi, China.*

[2] *Department of Occupational and Environmental Health and the Ministry of Education Key Lab of Hazard Assessment and Control in Special Operational Environment, Fourth Military Medical University, Xi'an, Shaanxi, China.*

[3] *Department of Physics, Institute of Photonics and Photon-Technology, Northwest University, Xi'an, Shaanxi, China.*

[#]These authors contributed equally to this work.

[*]Corresponding author: Zhe Wang (E-mail: wangzhe@fmmu.edu.cn) and Xue Yu Hu (E-mail: huxueyu@fmmu.edu.cn).

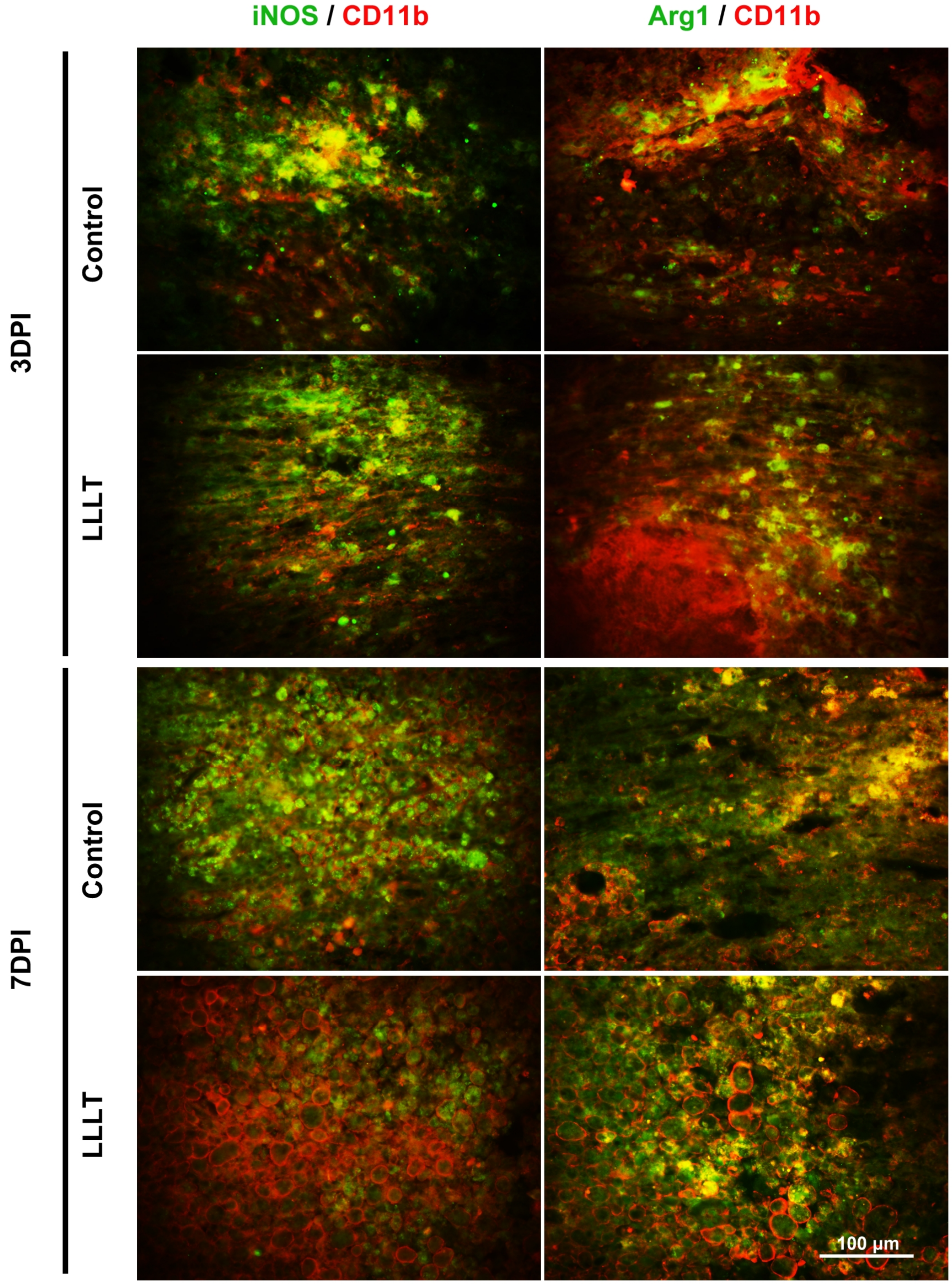

Fig. S1 Polarization of macrophage/microglia at the epicenter at 3dpi and 7dpi

Supplementary Figure 1 Polarization of macrophage/microglia at the epicenter at 3 and 7 dpi. $iNOS^+$ $CD11b^+$ cells refer to M1 phenotype, $Arg1^+CD11b^+$ cells refer to M2 phenotype. Positive cells in the merged panels exhibit a yellow signal. iNOS, inducible nitric oxide synthase; Arg1, arginase 1. 3DPI and 7DPI refer to 3 and 7 days post-injury, respectively. Bar = 100 μm.

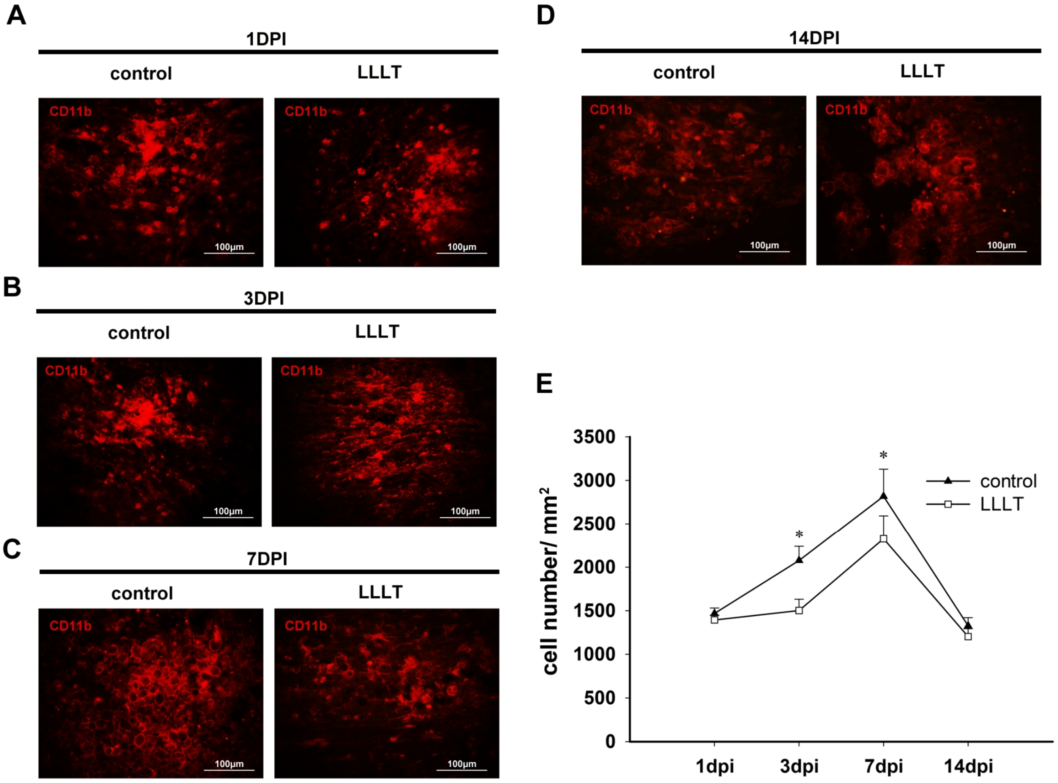

Fig.S2 Time course of macrophage/microglia activation

Supplementary Figure 2 Time course of macrophage/microglia activation. (A-D) Representative images of activated macrophage/microglia stained with CD11b. **(E)** Quantification of $CD11b^+$ macrophage/microglia cell numbers. Cell numbers were normalized as average number per mm^2 cord section, then quantitatively analysed. 1DPI, 3DPI, 7DPI, and 14DPI refer to 1, 3, 7, and 14 days post-injury, respectively. Bar = 100 μm; (2 measurements per section and 2 sections per rat with 3 rats per group; *, $P < 0.05$, compared with control).
